# Iron Deposition and Ferritin Heavy Chain (Fth) Localization in Rodent Teeth

**DOI:** 10.1186/1756-0500-6-1

**Published:** 2013-01-02

**Authors:** Xin Wen, Michael L Paine

**Affiliations:** 1 Center for Craniofacial Molecular Biology, Herman Ostrow School of Dentistry, University of Southern California, Los Angeles, USA

**Keywords:** Amelogenesis, Enamel, Endosomes, Ferritin, Immunohistochemistry, Iron

## Abstract

**Background:**

An iron rich layer on the labial surface is characteristic of the enamel of rodent incisors. In order to address a role for iron content in continuously growing incisors during odontogenesis, we studied iron deposition patterns in enamel and dentine using Perls’ blue staining and ferritin heavy chain (Fth) immunolocalization. Fth expression is regulated by iron level; therefore its localization can be used as a sensitive indicator for iron deposition.

**Results:**

Sagittal sections of 4-week old rat incisors showed a gradual increase in iron level in the enamel organ from secretory to maturation stages. In addition, iron was detected in ameloblasts of erupting third molars of 4-week old rats, suggesting iron plays a role in both incisor and molar development. In odontoblasts, the presence of iron was demonstrated, and this is consistent with iron’s role in collagen synthesis. Using postnatal 3-, 6-, 9-day old mice, the spatial and temporal expression of Fth in tooth development again indicated the presence of iron in mature ameloblasts and odontoblasts.

**Conclusions:**

While these data do not explain what functional role iron has in tooth formation, it does highlight a significant molecular activity associated with the formation of the rodent dentition.

## Background

Rodent incisors are characterized by yellowish pigmentation at labial side due to the presence of iron, with an iron content of about 0.030% in the whole upper incisors and 0.027% in the whole lower incisors [1]. Electron microscopy has shown that iron is found only in the region of the enamel organ associated with maturation [2]. The function of iron in enamel is not understood. A quantitative analysis on butterflyfish *Chaetodon miliaris* teeth found that those feed on harder prey have more iron than those that feed on softer-bodied prey, suggesting that iron serves as a strengthening agent to resist abrasion and cracking [3], and this is equally feasible in rodent incisor teeth. Furthermore, the iron concentration is inversely related to the level of calcium in the lingual edge of the tooth cap of butterflyfish [4], consistent with earlier observations that rats with a diet high in calcium showed decreased iron pigmentation in enamel [5] while incisors of iron deficient rats showed higher calcium content in outer enamel [6]. This also suggests that iron and calcium may be able to reversibly substitute for each other in hydroxyapatite. It has also been proposed that iron can decrease the solubility of crystallized hydroxyapatite because iron density positively correlates with acid-resistance of outer enamel [7]. In addition, many knockout or transgenic animals targeting the silencing or overexpression of enamel gene products result in an enamel with a chalky white appearance and structural defects, suggesting the incorporation of iron into enamel is linked to the normal process of enamel formation [8,9].

Iron is essential to all living organisms. The most abundant iron-containing proteins are hemoproteins that are involved in oxygen transport and delivery. In addition, iron’s ability to shuttle between ferric iron (Fe^3+^) and ferrous iron (Fe^2+^) makes it especially useful in electron transport and enzyme catalysis. By the same token, unregulated iron can cause cellular damage by catalyzing reactions leading to the production of toxic oxygen radicals [10,11]. Excess iron that is not for immediate use is stored in ferritin, a shell-like structure with a central, Fe^3+^ containing, cavity. Mammalian ferritins are 24-subunit heteropolymers made of two different subunit types, a heavy and light chain, coded by *Fth* and *Ftl* genes respectively. The early embryonic lethality in *Fth* knockout mice suggests an critical role for ferritin during organismal development [12]. The expression of Fth and Ftl is post-transcriptionally regulated by iron level [13]. When cellular iron levels are low, the iron regulatory proteins IRP1 and IRP2 bind to iron responsive elements, IREs, located in the 5’ untranslated region of the *Fth* and *Ftl* mRNA, and block the translation initiation of both genes. When iron levels are high, the iron-bound IRPs dissociate from the mRNA, thereby allowing translation of Fth and Ftl to proceed [14,15]. Given the high iron content in mature enamel, not surprisingly, *Fth* was identified as one of the genes most highly up-regulated in maturation ameloblasts when compared to secretory ameloblasts [16]. Earlier electron microscopic studies have also shown that ferritin is present only in maturation ameloblasts and papillary layer, but not in secretory ameloblasts [2,17].

Iron also functions as a cofactor of prolyl hydroxylase, which catalyzes formation of hydroxyl proline, a key step in collagen’s triple helix formation [18]. Since collagens comprise of 90% of dentin extracellular matrix molecules [19], iron is presumably present in odontoblasts for producing collagen. However, few studies have shown the presence of iron in odontoblasts, probably due to much lower iron level when compared to that in ameloblasts, and also the low sensitivity of iron staining method. Based on the knowledge that the amount of ferritin responds to iron levels [13], the presence of iron in odontoblasts was implied with immunolocalization of ferritin in this study.

Published reports on the presence of iron and ferritin in teeth have mainly been limited to observations in ameloblasts and in the enamel of rodent incisors [2,20]. Iron uptake in developing rat molars has been observed with autoradiographic methods [21]. In the present study, the increasing iron deposit and ferritin expression in the enamel organ cells of rat incisors, throughout amelogenesis, is demonstrated. Additional data are also presented to illustrate the presence of iron in ameloblasts of molar teeth prior to eruption. The spatiotemporal expression profiles of Fth throughout incisor and molar tooth development are also shown using postnatal 3-, 6-, 9-day old mouse samples.

## Methods

### Section preparation

All vertebrate animal manipulation complied with Institutional and Federal guidelines. Approval was given through the University of Southern California Institution’s Animal Care and Use Committee (IACUC), protocol # 11736. The 4-week old female Swiss Wistar rat was first put into a glass jar supplied with ether-soaked paper and followed by intraperitoneal injection with sodium pentobarbital at about 5 mg per 100 gram of rat body weight. After the lack of reflex was confirmed by paw pinch, an incision was made through abdomen along the length of the diaphragm. Two parallel cuts were made from the bottom of the diaphragm toward the head across ribs. The end of the sternum was grasped and flipped to expose the heart. Cannula tip was inserted through the left ventricle into the ascending aorta. The right atrium was pierced to allow the escape of return circulation. The pump was set at 10 ml/min. After a brief phosphate-buffered saline (PBS) flush, perfusion line was switched to fixative (4% paraformaldehyde, 0.1% glutaraldehyde, 0.08M sodium cacodylate, 0.05% calcium chloride, pH7.2-7.4). Perfusion was complete when the spontaneous movement of the tail or paws (‘formalin dance”) was observed (about 50-100 ml of fixative per rat). Jaws were dissected and immersed in fixative overnight at 4°C. The samples were washed with PBS and decalcified in 4.13% disodium ethylenediaminetetraacetic acid (EDTA) for about 2 months at 4°C with frequent change of decalcification solution. The samples were embedded in paraffin and 4 mm sections were cut with microtome.

The postnatal 3-, 6-, 9-day old Swiss Webster mice were sacrificed and jaws were dissected and fixed by immersion in 4% paraformaldehyde overnight and decalcified in 10% EDTA for 2 weeks. Embedding and cutting are the same as the rat samples.

### Perls’ blue staining

Perls’ reagent, potassium ferrocyanide, in the presence of hydrogen chloride, can react with Fe^+3^ to form an insoluble pigment known as Prussian blue. Paraffin sections were deparaffinized, rehydrated, and immersed in Perls’ blue staining solution, which was freshly prepared immediately before use by mixing equal parts of 20% hydrochloric acid (20% of concentrated HCl) and 10% potassium ferrocyanide, for 40 minutes [22]. The samples were then washed with distilled water, counterstained with nuclear fast red, dehydrated with graded alcohol series, cleared with xylene and mounted with cover slips. For 3,3-diaminobenzidine (DAB)-H_2_O_2_ enhanced staining, sections were further incubated with freshly mixed 0.5 mg/ml DAB and 0.02% H_2_O_2_ solution for about 5-10 minutes [23].

### Immunohistochemistry

Paraffin sections were deparaffinized and rehydrated. The endogenous peroxidase activity was quenched by 3% H_2_O_2_ in methanol for 10 minutes. Anti-Fth1 antibody (LS-B5847) was obtained from LifeSpan Biosciences (Seattle, WA) and used at 1:200 dilution in 1% BSA-0.5% Triton X-100-PBS solution. ImmPRESS reagent anti-rabbit Ig peroxidase (MP-7401) and ImmPACT DAB peroxidase substrate (SK-4105) were purchased from Vector laboratories (Burlingame, CA) and instructions were followed except for the washing buffer is 0.1% Triton X-100-PBS solution throughout the staining.

## Results

### Detection of iron and ferritin heavy chain (Fth) in the rat and mouse dentition

Below are presented a series of images indicating the spatiotemporal expression of iron and Fth in rats (Figures 1, 2, 3, 4, 5 and 6) and mice (Figures 7 and 8).

**Figure 1:**
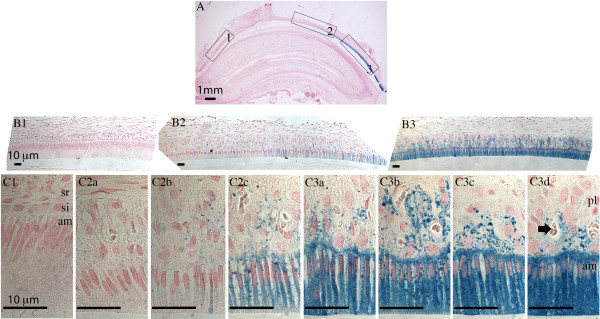
**Perls’ blue staining of rat maxillary incisor.** Sagittal section of 4-week old rat maxillary incisor was stained with Perls’ Prussian blue for detection of iron and counterstained with nuclear fast red. **Panel A:** Whole incisor observed under stereoscope with 12.5 x magnification. **Panels B1 - B3:** Boxed regions (1-3) in panel **A** are shown with 100x magnification. **Panels C1, C2a - 2c, and C3a - 3d:** Progression from secretory stage amelogenesis (**C1**) to late maturation stage amelogenesis (**panel C3d**) with 630x magnification. Enamel organ cells (ameloblasts, stratum intermedium, stellate reticulum and papillary layer cells) are labeled in panels **C1** and **C3d**. The arrow in panel **C3d** shows a blood vessel surrounded by papillary layer cells. The following abbreviations are used: am, ameloblasts; si, stratum intermedium; sr, stellate reticulum; pl, papillary layer. Scale bars: **A**, 1 mm; **B1-B3** and **C1-C3d**, 10 μm.

**Figure 2:**
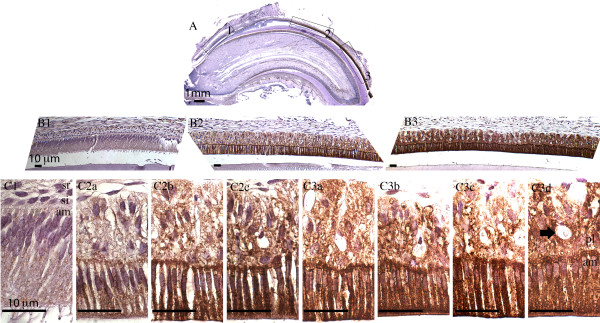
**Immunohistochemical staining of Fth in maxillary incisor.** Sagittal section of 4-week old rat maxillary incisor was stained with anti-Fth antibody and counterstained with hematoxylin. **Panel A:** Whole incisor observed under stereoscope with 12.5x magnification. **Panels B1-B3:** Boxed regions (1-3) shown in panel **A** are shown with 100x magnification. **Panels C1, C2a - 2c and C3a - 3d:** Progression from secretory stage amelogenesis (**panel C1**) through late maturation stage amelogenesis (**panel C3d**) with 630x magnification. Late stage ameloblasts and the overlying papillary layer cells are the most highly staining cells in this progression. Enamel organ cells (ameloblasts, stratum intermedium, stellate reticulum and papillary layer cells) are labeled in panels **C1** and **C3d**. The arrow in panel **C3d** shows a blood vessel surrounded by papillary layer cells. The following abbreviations are used: am, ameloblasts; si, stratum intermedium; sr, stellate reticulum; pl, papillary layer. Scale bars: A, 1 mm; **B1**-**B3** and **C1**-**C3d**,10 μm.

**Figure 3:**
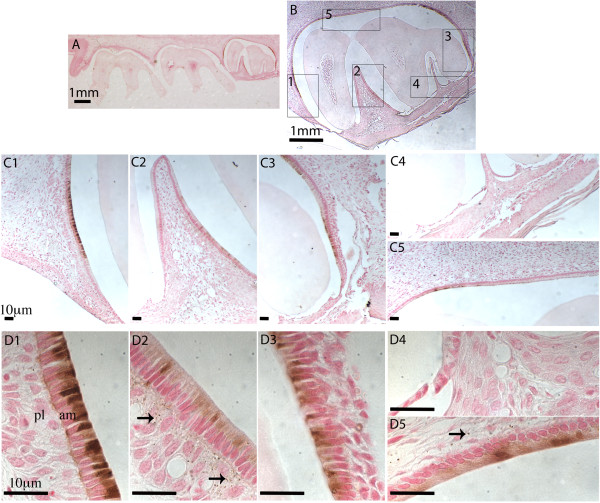
**Iron detection in the rat maxillary third molar.** Sagittal section of 4-week old rat maxillary molars were stained with DAB enhanced Perls’ blue for iron and counterstained with fast red. **Panel A:** Section of three molars observed under stereoscope with 16x magnification. **Panel B:** The third molar shown with 40x magnification. **Panels C1 - C5:** Regions 1 - 5 as identified in panel B are shown with 100x magnification. **Panels D1 - D5:** Ameloblasts and papillary layer cells, corresponding again to regions 1 - 5 as identified in panel **B**, are shown with 630x magnification. Ameloblasts (am) and papillary layer cells (pl) are labeled in panel **D1**. The arrows in **D2** and **D5** show iron deposits in papillary layer cells. Scale bars: **A** and **B**, 1 mm; **C1**-**C5** and **D1**-**D5**, 10 μm.

**Figure 4:**
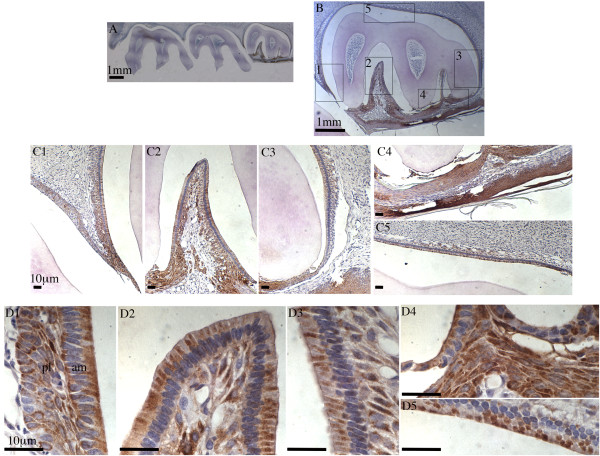
**IHC staining of Fth in rat maxillary third molar.** Sagittal section of 4-week old rat maxillary molars stained with anti-Fth antibody and counterstained with hematoxylin. **Panel A:** Section of first, second and third molars observed under stereoscope with 16x magnification. **Panel B:** The third molar shown with 40x magnification. **Panels C1 - C5:** Regions identified as 1 - 5 in panel **B** are shown with 100x magnification. **Panels D1 - D5:** Ameloblasts and papillary layer cells, corresponding again to regions 1 - 5 indicated in panel **B**, are shown with 630x magnification. Ameloblasts (am) and papillary layer cells (pl) are labeled in panel **D1**. Scale bars: A and B, 1 mm; C1-C5 and D1-D5, 10 μm.

**Figure 5:**
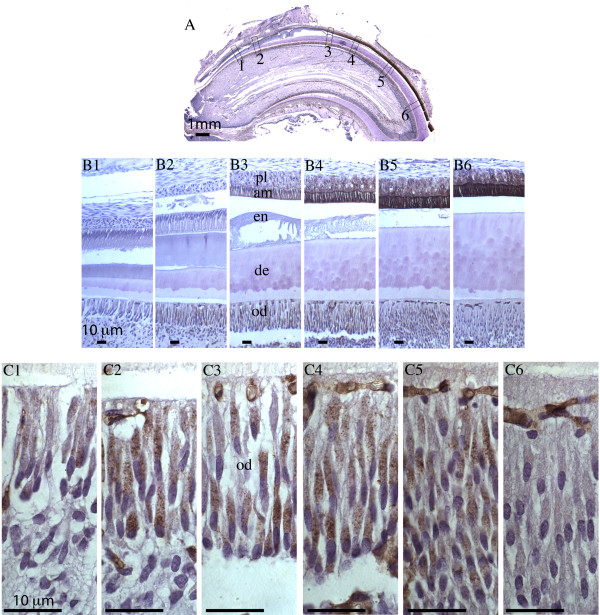
**IHC staining of Fth in odontoblasts of rat maxillary incisor.** Sagittal section of 4-week old rat maxillary incisor was stained with anti-Fth antibody and counterstained with hematoxylin. **Panel A:** Whole incisor observed under stereoscope with 12.5x magnification. **Panels B1 - B6:** Boxed regions (1 - 6) identified in panel **A**, spanning from outer enamel epithelia to odontoblasts, are shown with 100x magnification. **Panels C1 - C6:** Odontoblasts corresponding to the indicated regions 1 - 6 in panel **A** are shown with 630x magnification. Papillary layer cells (pl), ameloblasts (am), enamel (en), dentin (de), and odontoblasts (od) are labeled in panels **B3** and **C3**. Scale bars: **A**, 1 mm; **B1-B6** and **C1-C6**, 10 μm.

**Figure 6:**
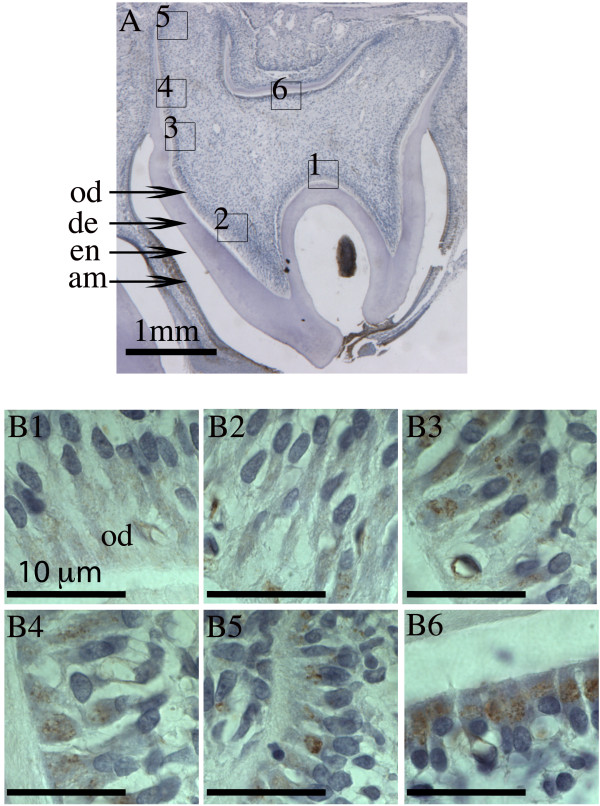
**IHC staining of Fth in odontoblasts of rat maxillary third molar.** Sagittal section of a 4-week old rat maxillary third molar stained with anti-Fth antibody and counterstained with hematoxylin. **Panel A:** the third molar observed under stereoscope with magnification 40x. **Panels B1 - B6:** Odontoblasts corresponding to indicated regions 1 - 6 identified in panel **A** are shown with 630x magnification. Ameloblasts (am), enamel (en), dentin (de), and odontoblasts (od) are labeled in panels **A** and **B1**. Scale bars: **A**, 1 mm; **B1-B6**, 10 μm.

**Figure 7:**
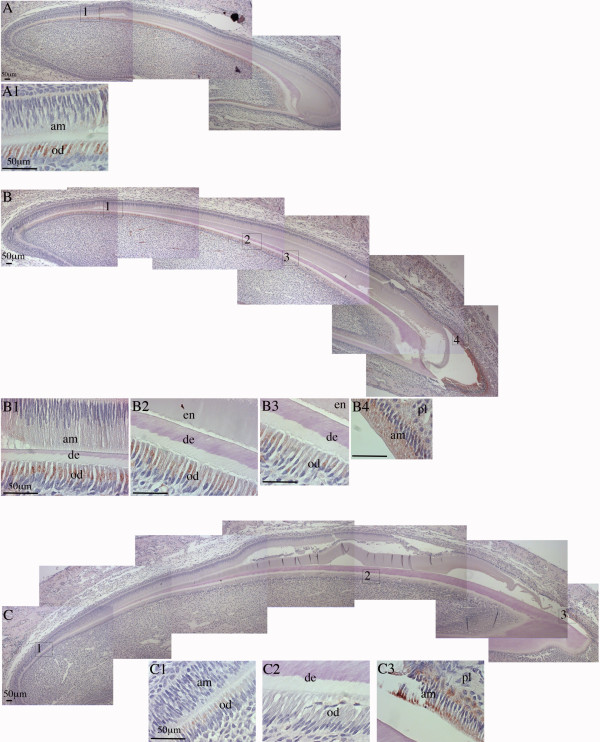
**IHC staining of Fth in mouse mandibular incisors.** Sagittal section of postnatal (PN) 3, 6, and 9-day old mouse mandibular incisors were stained with anti-Fth antibody and counterstained with hematoxylin. **Panel A:** PN3 incisor observed with 100x magnification. Three pictures were overlapped to show the whole incisor. **A1,** box 1 in **A** shown with 630x magnification. **Panel B:** PN6 mandibular incisor observed with 100x magnification. Six pictures were overlapped to show the whole incisor. **B1-B4,** boxes 1-4 in **B** shown with 630x magnification. **Panel C:** PN9 mandibular incisor observed with 100x magnification. Seven pictures were overlapped to show the whole incisor. **C1-C3,** boxes 1-3 in **C** shown with 630x magnification. Papillary layer cells (pl), ameloblasts (am), enamel (en), dentin (de), and odontoblasts (od) are labeled. Scale bars: 50 μm.

**Figure 8:**
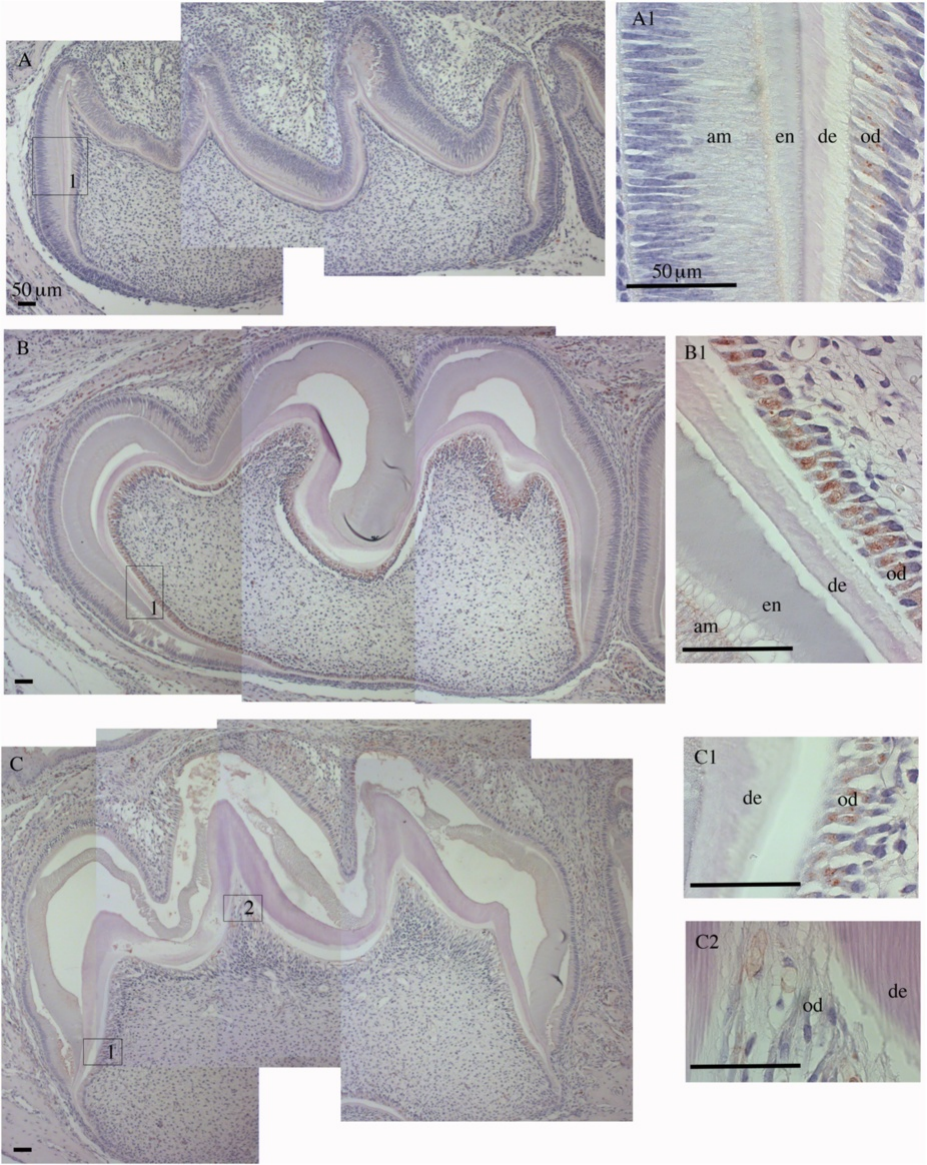
**IHC staining of Fth in mouse mandibular first molars.** Sagittal section of postnatal (PN) 3, 6, and 9-day old mouse mandibular first molars were stained with anti-Fth antibody and counterstained with hematoxylin. **Panel A:** PN3 mandibular first molar observed with 100x magnification. Three pictures were overlapped to show the whole molar. **A1,** box 1 in **A** shown with 630x magnification. **Panel B:** PN-6 mandibular first molar observed with 100x magnification. Three pictures were overlapped to show the whole molar. **B1,** box 1 in B shown with 630x magnification. **Panel C:** PN-9 mandibular first molar observed with 100x magnification. Four pictures were overlapped to show the whole molar. **C1 and C2,** boxes 1 and 2 in **C** shown with 100x magnification. Ameloblasts (am), enamel (en), dentin (de), and odontoblasts (od) are labeled. Scale bars: 50 μm.

### Iron detection in rat incisors using Perls’ blue staining

The maxillary incisors of 4-week old rats were stained with Perls’ blue for iron deposits in the tissues. Sagittal sections of incisor showed a gradual increase in iron level in the enamel organ from secretory to maturation (Figure 1). No Perls’ blue was detected in early secretory ameloblasts or the overlying cells of the stratum intermedium, stellate reticulum or outer dental epithelium (Figure 1, panel B1 and C1). Perls’ blue precipitates gradually accumulated in the early maturation ameloblasts and the overlying papillary layer cells (Figure 1, panel C2b) until very high staining was noted in late maturation ameloblasts, and to a lesser extent, in the overlying papillary layer cells (Figure 1, panels C3b – C3d). These data suggested iron was transported from the circulatory system to papillary layer cells and ameloblasts to be eventually deposited to the forming enamel matrix as the outer enamel layer mineralizes.

### Immunolocalization of the Fth protein in rat incisors

Iron is involved in redox reactions and high level of iron leads to the over-production of oxygen reactive species, which can be toxic to cells. One of the mechanisms to regulate iron level is to increase the expression of the iron storage protein ferritin. Immunohistochemistry staining with anti-Fth antibody showed that ferritin expression (Figure 2) and iron level (Figure 1) were clearly correlated throughout the developmental course of the rodent incisor. Fth expression was barely detectable or absent in the secretory stage ameloblasts (Figure 2, panel C1) and started to appear in the first of the ruffle-ended ameloblasts (RA) (Figure 2, panel C2a). With progression to late maturation stage, the intensity of immunoreactivity increased and most notably in ameloblasts and papillary layer cells. This result was consistent with a previous study, in which *Fth* was identified as one of the upregulated genes during enamel maturation when compared to secretory stage [24].

### Iron detection in ameloblasts of erupting rat molars

When 4-week old rat maxillary molars were stained with the Perls’ reagent, a weak blue coloration developed in mature ameloblasts of the unerupted third molar (data not shown). To enhance the sensitivity, DAB and H_2_O_2_ were subsequently used to react with Perls’ blue to form dark brown coloration [23]. Only ameloblasts of the unerupted third molar showed iron deposits as indicated by brown coloration (Figure 3). The more mature ameloblasts located adjacent to the second molar showed the strongest staining (Figure 3, panels C1 and D1). The regions between the cusps showed lower levels of iron deposition in ameloblasts (Figure 3, panels C2 and D2). The overlying papillary layer cells also showed iron deposit, but only in regions where the iron level was relatively low in proximal ameloblasts (Figure 3, panels D2 and D5). As iron levels increased in more mature ameloblasts, the immediately overlying papillary layer showed little iron deposit (Figure 3, panel D1). Taken together, these results suggested the pathway for iron movement was from the papillary layer to ameloblasts, and this was consistent with what was observed in incisors.

Figure 4 showed a series of panels, similar to those presented in Figure 3, of mandibular molar cells with Fth immunostaining of the unerupted third molar. Here Fth was clearly expressed in ameloblasts in a similar spatiotemporal profile to that seen for iron using Perl’s, DAB and H_2_O_2_. The expression of Fth was also more clearly evident in the papillary layer cells, and this may be due to the higher sensitivity of immunostaining technique, and the fact that mobilization of ferritin iron proceeds ferritin degradation [25].

### Ferritin expression in rat odontoblasts

Dentin is a collagen-based hard tissue. Iron is required in collagen synthesis [18]. We have been unable to directly demonstrate any noticeable levels of iron concentration in odontoblasts using DAB-H_2_O_2_ intensified Perls’ staining (data not included). However, immunohistochemistry with Fth antibody has been used to indirectly examine the likely presence of iron in odontoblasts. In incisors, Fth can be initially detected in odontoblasts at very early developmental stage, prior to any appreciable expression in adjacent ameloblasts (Figure 5, panels B1, B2, C1, and C2). A similar level of Fth immuno-staining was also detected in odontoblasts located at the lingual side of the incisor (data not shown). Although the expression of Fth in odontoblasts developmentally preceded that in ameloblasts, the overall expression level was significantly lower than that in mature ameloblasts (Figure 5, panels B1 – B6).Fth was undetectable in odontoblasts when dentin formation significantly slowed and enamel had reached a full thickness prior to eruption (Figure 5, panel C6). The Fth expression in odontoblasts of these 4-week-old rats did not show a clear gradient similar to what had been observed in ameloblasts as they transitioned from secretory to maturation stage. Nevertheless, the expression patterning was consistent with the involvement of iron in collagen polymerization during dentin formation.

In third molar odontoblasts from 4-week old rats, a similar result was obtained regarding the expression of Fth (Figure 6). Fth can be detected in odontoblasts in root area (Figure 6 panels B4 and B5), and in odontoblasts of the more differentiated regions (Figure 6 panels B3 and B6). Fth could not be detected in odontoblasts at the tip and crown region which is where dentin formation had slowed, enamel formation was complete, and the tooth was about to erupt (Figure 6 panels B1 and B2).

### Ferritin expression in developing mouse mandibular incisor

In order to study spatial and temporal localization of iron in mouse tooth development, immunohistochemistry with Fth antibody was again used to indirectly illustrate the iron deposit pattern in postnatal (PN) 3-, 6-, and 9-day old mouse. Fth expression was detected in odontoblasts of PN3 incisors throughout the sagittal section (Figure 7, panel A), indicating iron presence in these odontoblasts that were actively synthesizing collagen. On the other hand, Fth expression was undetectable in ameloblasts of PN3 incisors, which was as expected, considering ameloblast secretory and maturation activities begin later than odontoblasts and our earlier results that iron deposition was only detected in mature ameloblasts in 4-week rat incisors. Fth expression in PN6 incisor showed a clear gradient with higher expression in odontoblasts near cervical loop where dentinogenesis were robust (Figure 7, panel B1) and lower expression in more mature regions where dentin almost reached full thickness (Figure 7, panel B3). At the tip of the PN6 incisor, ameloblasts started to show Fth expression (Figure 7, panel B4), reflecting the presence of iron in these mature ameloblasts. In just erupted and continuously growing PN9 incisors, the correlation of Fth expression with collagen-synthesizing odontoblasts and mature ameloblasts was more evident. Fth expression was only detected in odontoblasts at the growing end (Figure 7, panel C1) and ameloblasts at the incisal, or fully mature end (Figure 7, panel C3).

### Ferritin expression in developing mouse mandibular molar teeth

In PN3-, 6-, and 9-day old mouse mandibular first molars, Fth expression was detected in odontoblasts of all three samples (Figure 8). In PN3 molars, the expression of Fth (Figure 8, panel A) was less prominent than that in PN3 incisors (Figure 7, panel A), which was consistent with the notion that molars are developmentally delayed when compared to incisors. PN6 molars showed the strongest Fth expression in odontoblasts when compared to either PN3 or PN9 (Figure 8, panel B1). In PN9 molars, Fth expression was only seen in odontoblasts in the root area (Figure 8, panel C1) where dentinogenesis (root formation) was continuing, but not in odontoblasts corresponding to the cusp regions where dentin formation had slowed (Figure 8, panel C2). Not surprisingly, Fth expression was not detected in ameloblasts of these mouse molars, considering Fth expression in PN6 and PN9 incisors (Figure 7, panel B4 and C3) was only observed in ameloblasts at the very tip and the ameloblasts in PN6 and PN9 molars were at much less mature stage. Taken together, the Fth expression pattern in developing mouse teeth again showed iron was present in dentin forming odontoblasts and mature ameloblasts.

## Discussion

The literature discussing iron deposits on the labial surface in most rodent incisors can be dated over a century ago [26], in which hypoparathyroidism resulted in loss of pigment in rat incisors. Early studies also led to two main and seemingly contradictory ideas regarding the function of iron in enamel. Stein and Boyle [27] have concluded that pigmentation does not affect enamel structural properties based on the observation that after surgically destroying the pigment-containing part of the enamel organ, the integrity of the underlying enamel is not affected. On the other hand, Prime et al. [28] showed that prolonged iron deficiency caused loss of pigmentation as well as enamel hypoplasia and aplasia, suggesting iron deficiency is associated with severe structural defects of enamel. Our results that iron is not only present in ameloblasts of continuously growing incisors, but also evident in ameloblasts of molars, favor the idea that iron is an integral component for enamel formation. A prior study on incorporation of iron in rat incisor showed that iron is not released from the ameloblasts and deposited into the enamel until the calcium and phosphorus contents of the enamel have reached a maximum level [29]. In addition, there is an inverse relationship between the iron and calcium content in the outer enamel layer [30]. The hypothesis proposed by Halse and Selvig (1974) is that enamel mineralization advances only to a point, leaving room for subsequent incorporation of iron accompanied by removal of calcium. Therefore, iron incorporation may represent the final refinement for enamel mineralization to possibly provide extra strength or acid resistance. Although iron deposit in incisor enamel is evident, further studies on the mineral content of molar enamel would be required to confirm the idea.

Iron is actively involved in numerous biological functions by serving as a cofactor for many proteins, including hemoglobins in oxygen binding and transport, catalases and peroxidases in oxygen metabolism, cytochromes in oxidative phosphorylation and in electron transport. [31]. Energy-requiring events, such as active ion transport to, and water and matrix protein removal from the maturing enamel in maturation stage ameloblasts, demand for high level of adenosine triphosphate (ATP) production through oxidative phosphorylation in mitochondria [32]. Therefore, it is conceivable that iron is needed in mature ameloblasts to assist energy production needs. Iron is also required in dentin formation. It is known that the maturation of collagen, the major protein in dentin, requires hydroxylation of proline residues in –X-Pro-Gly- sequences in order to form stable triple helical structure under physiological condition [33]. Prolyl 4-hydroxylase, the enzyme catalyzing the hydroxylation of prolines, in turn requires ascorbate, iron and oxygen as cofactors [18]. Therefore, iron is indispensable for the formation of dentin. It has been shown that tetracycline, an iron chelator, severely inhibits the development of *in vitro* cultured E15 incisor while iron can rescue the inhibition [34]. Consistent with this finding, using Fth antibody, we have showed indirectly the presence of iron in odontoblasts, presumably involved in collagen formation. Furthermore, iron is only detected in odontoblasts that are actively involved in dentinogenesis. Our results also show that iron deposition in odontoblasts occurs much earlier than that in adjacent ameloblasts, consistent with the fact that dentinogenesis is initiated prior to enamel formation.

We also noticed that the presence of iron in mature ameloblasts seems to be a phenomena linked to the erupting nature of the tooth. In 4-week old rat molars, iron is present in enamel organs of pre-erupting third maxilla molars. Consistently, iron deposit was observed in pre-erupting first maxilla molar when 8-day old rat molars were examined [21]. Moreover, the strong iron accumulation in rodent incisors match well with the continuously erupting nature. It would be interesting to further study the possible involvement of iron in tooth eruption.

## Conclusions

In conclusion, we have systematically showed iron deposit in both ameloblasts and odontoblasts, in both incisors and erupting molars of 4-week old rats, and in different tooth developmental stages in postnatal 3-, 6-, and 9-day old mice. Our data on Fth expression and iron deposition support the idea that iron is actively involved in tooth development.

## Abbreviations

Fth: Ferritin heavy chain; Ftl: Ferritin light chain; PBS: Phosphate-buffered saline; DAB: 3,3-diaminobenzidine (DAB); PN: Post natal; ATP: Adenosine triphosphate; EDTA: Ethylenediaminetetraacetic acid.

## Competing interests

The authors declare that they have no competing interests.

## Authors’ contributions

XW and MP conceived and designed the experiments; XW performed all the experiments; XW and MP analyzed the data and wrote the paper. Both authors have critically read, edited and approved the final manuscript. XW and MP accept full responsibility for the integrity of the data analysis.
